# Use of biologics in allergen immunotherapy 

**DOI:** 10.5414/ALX02206E

**Published:** 2021-02-19

**Authors:** Wolfgang Pfützner, Mathias Schuppe

**Affiliations:** Department of Dermatology and Allergology, Clinical-Experimental Allergology, Allergy Center Hessen, University Hospital Marburg, Philipps-University Marburg

**Keywords:** biologics, allergen-immunotherapy, omalizumab, food allergy, insect venom allergy, allergen blocking antibodies

## Abstract

Biologics are drugs that are derived or synthesized from biological sources. A particular class are recombinant monoclonal antibodies. Their targeted application against distinct molecules of intercellular communication is of significant relevance in the treatment of tumor, inflammatory, or allergic diseases. But also in the context of allergen immunotherapy (AIT) they can be of special value. This is exemplified by the anti-IgE antibody omalizumab, which allows to achieve allergen tolerance in patients suffering from severe allergic reactions and increased risk of AIT-induced anaphylaxis. Furthermore, omalizumab administration during AIT effectively lowers the rsik of allergic side effects. This is demonstrated by a variety of studies and case reports of patients suffering either form respiratory, food, or insect venom allergy. Besides a direct blocking of IgE-mediated effects, T-cellular immune mechanisms might also be involved. Another interesting option is the applcation of recombinant IgG antibodes directed against specific epitopes of an allergen. Similar to AIT-induced IgG antibodies they can prevent the binding of allergens to IgE-antibodes as well as the hereby elicited allergic reactions.

## Introduction 

Biologics are defined as drugs derived from biological sources and as such are also referred to as biopharmaceuticals. A variety of organisms can be considered as sources, for example human cell lines and genetically modified cells, microorganisms such as yeast cultures or (*Escherichia coli*) bacteria, but also animals such as mice, rats, rabbits for the production of antibodies and immune sera or pigs and cattle for the production of insulin. Even humans “produce” biologics, which are used in the form of cellular products (for example, red cell or platelet concentrates) or proteins (plasma, clotting factors, intravenous immunoglobulins (IVIG)). Whole blood can be considered as one of the oldest biologics, which was already drunk or rubbed in in ancient times as a strength and youth-giving agent, before it was used for transfusions after the discovery of the blood circulation by William Harvey (1578 – 1657) [1]. 

Considering these defining characteristics, it can be stated that biologics also form the basis of allergen immunotherapy (AIT). Thus, therapeutics derived from “living” allergen sources, such as pollen extracts or insect venoms, constitute biopharmaceuticals in the same way as porcine insulin or human albumin. However, these allergen preparations will not be the subject of the following discussion. Instead, the focus will be on antibodies, which are usually referred to in today’s linguistic usage as “biologics”, and which are also classified as biomodulators (“biological response modifiers”) due to their functional property of specifically binding distinct protein components and thereby specifically intervening with biological, generally immunological, processes. 

## IgG anti-IgE antibodies: omalizumab 

**Figure 1:**
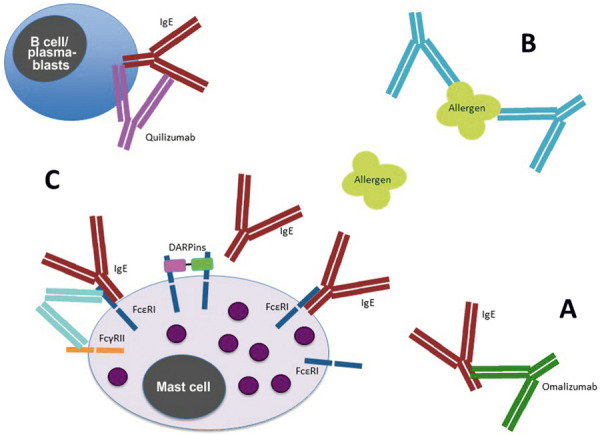
Use of biologics in allergen immunotherapy. At different levels, allergen-mediated activation of IgE-bearing cells can be prevented, thus supporting (A, C) or even substituting (B) the implementation of AIT. A: The anti-IgE antibody omalizumab (as well as ligelizumab, currently being tested in phase III trials) binds free IgE and can thus prevent its fixation via the high-affinity IgE receptor FcɛRI on effector cells such as mast cells and basophils (or the low-affinity FcɛRII on denritic and B cells). B: Epitope-specific IgG monoclonal antibodies can complex allergen, preventing its binding to IgE. C: The IgG antibody quilizumab binds membrane-bound IgE on B cells and plasmablasts resulting in their depletion; DARPins not only prevent IgE fixation on FcɛRI but can also promote its dissociation; FcɛRI inhibitors inhibit IgE receptors via activation of the inhibitory receptor FcγRII.

Omalizumab is a humanized IgG1K monoclonal antibody produced in an ovarian Chinese hamster cell line. It is marketed under the compound name Xolair (Novartis, Basel, Suisse) and is approved for the treatment of allergic bronchial asthma (AB) and chronic spontaneous urticaria [2]. The antibody binds the CH3 domain of the IgE heavy chain, preventing its fixation by the high-affinity IgE receptor FcɛRI (Figure 1B). In addition, there is a decrease in free IgE in serum due to the formation of omalizumab-IgE complexes, which are eliminated hepatically, and a subsequent downregulation of FcɛRI on basophils and mast cells [3, 4]. These effects prevent IgE-mediated responses, resulting in decreased release of histamine and other inflammatory mediators from mast cells and basophils. Clinically, this leads to substantially improved disease control in asthmatics and urticaria patients as well as prophylaxis of anaphylactic reactions in off-label use in mastocytosis [2]. 

The rationale for the use of omalizumab in AIT is based on these principles of its mode of action [5]. 

Primary goals are

- achieving AIT-mediated tolerance in patients in whom an increase of the allergen dose during the initial up-dosing phase would otherwise not be possible due to their pronounced IgE-mediated immunoreactivity,
- and minimization of the risk of AIT-related allergic side effects.

Several studies have investigated the benefit of omalizumab regarding these objectives, looking at AITs with aeroallergens, food or insect venoms, in the form of prospective placebo-controlled or open-label trials as well as retrospective analyses and casuistic reports. 

## Omalizumab in AIT with aeroallergens

In several double-blind, placebo-controlled (DBPC) studies, omalizumab was used as an adjunctive medication in patients with respiratory allergies receiving AIT, with study subjects suffering from either allergic rhinitis (AR) alone, AB, or AR with additional AB. For example, in one DBPC study, 159 adult AR patients received omalizumab for 9 weeks before rush AIT with ragweed was initiated, also in a placebo-controlled manner, and continued in the following 12 weeks during the maintenance phase. Co-medication of omalizumab and AIT resulted in a significantly higher decrease of seasonal symptoms [6]. In a previous study with 221 children and adolescents with AR who received subcutaneous AIT with birch or grass pollen extract, omalizumab or placebo was administered in addition to AIT for a total of 24 weeks, after a 12-week induction phase and before the onset of the next pollen season. Omalizumab administration caused a significant symptom reduction in the birch (by 50%) or grass pollen season (by 57%) compared to the placebo-treated AIT cohort [7]. These results suggest that omalizumab has a positive effect especially in patients who are still hampered by increased clinical symptoms in the first pollen season under AIT because of a delayed tolerance induction. However, the studies do not allow conclusions on whether omalizumab administration is associated with a longer lasting benefit in terms of facilitated tolerance development, as there were no later follow-ups. 

More informative was a DBPC study of 140 adolescents and adults with seasonal AR and additional AB who were treated with grass pollen AIT. The subjects received the anti-IgE antibody 2 weeks before AIT initiation and then for 16 additional weeks, with the last 8 weeks comprising the pollen season. Patients experienced a significant reduction in seasonal symptoms (39% lower than placebo control) [8]. However, in the 2 subsequent years of continued AIT, there was no difference between individuals on the omalizumab and those on placebo co-medication [9], demonstrating no long-term or disease-modifying effect. Of the asthmatics, only those with moderate to severe AB showed improvement in pulmonary parameters, indicating that omalizumab has specific benefits in selective groups of more severely affected patients. This was confirmed in a large DBPC study of 248 adults with at least moderate persistent AB inadequately controlled by inhaled corticosteroids [10]. After a 13-week pretreatment with omalizumab or placebo, AIT with different aeroallergens (house dust mite, cat, or dog) was performed. Patients treated with omalizumab showed a significantly lower asthma score before the start of AIT and were significantly more likely to reach the target maintenance dose (almost 90% of them) compared to the placebo group; the main reason for discontinuation of study participation was systemic reactions to allergen administration. Severe asthmatic reactions occurred more frequently in placebo-treated individuals (24 vs. 6 omalizumab patients), particularly during the initiation phase of dose escalation (13 vs. 3). However, adverse events requiring epinephrine administration occurred also in the omalizumab cohort, although much less frequently, with 9 vs. 22 placebo patients. Likewise, in another study of AR individuals, fewer systemic symptoms were seen in the omalizumab-treated patients [7], whereas no difference was noticed in this regard between the two treatment groups of patients with AR ± AB in the study by Kopp et al. [9]. The effect of omalizumab on the development of AIT-related local reactions was also divergent, with a decreased frequency in some but not all studies (Table 1) [6, 9, 11]. 

## Omalizumab in AIT with food 

Unlike allergies to various aeroallergens and insect venoms, AIT for food allergies is still primarily an experimental treatment. Only formulation for the treatment of peanut allergy was approved last year by the FDA as oral AIT in children and adolescents aged 4 – 17 years [12]. Due to often pronounced anaphylactic reactions and the difficulty to reliably avoid triggering allergens, especially if they are hidden in manufactured products, AIT is of particular importance for food-allergic patients [13]. However, in comparison to the established forms of AIT, hypersensitivity reactions in the form of gastrointestinal complaints, asthma, or anaphylaxis occur significantly more frequently than in respiratory and hymenoptera venom allergies so that AIT must be discontinued or only tolerance of very low allergen doses is achieved [14]. Utilization of omalizumab therefore appears particularly suitable for oral AITs with food allergens [15]. Studies on this have been performed with peanut, cow’s milk, chicken egg, and allergen mixtures. 

In an open-label pilot study, 13 peanut-allergic patients aged 7 – 15 years, who had previously experienced anaphylactic reactions to 1 – 100 (median 50) mg of peanut, received 12 weeks of pre-treatment with omalizumab, which was continued during a subsequent 8-week AIT up-dosing phase with peanut flour up to a maximum daily dose of 4,000 mg [16]. Continuation of AIT at this dose for an additional 10 – 12 weeks was followed by DBPC provocation of a cumulative amount of 8,000 mg, which was equivalent to ~ 20 peanuts. All but 1 patient achieved the maintenance dose (92%) and ultimately tolerated peanut provocation. In a subsequent DBPC study, 37 peanut allergic patients aged 6 – 19 years were administered omalizumab (or placebo) for 12 weeks before initiating peanut AIT, up to a maximum maintenance dose of 2,000 mg peanut protein [17]. Maintenance therapy was then continued for 12 weeks without co-medication with omalizumab. While 75% of the placebo-treated patients already failed to reach the first increment of 250 mg peanut protein, this affected only 7.4% in the omalizumab group. After switching to non-blinded application of omalizumab, all could be dosed up to 2,000 mg peanut protein. Overall, 75.9% of the omalizumab-treated patients but only 12.5% of the placebo-treated patients tolerated oral provocation with 4,000 mg peanut protein afterwards. 

Somewhat divergent results are found in the studies of oral AIT with cow’s milk. Five children with previous unsuccessful cow’s milk AIT received renewed oral AIT in an open-label study after a 9-week pre-treatment with omalizumab, which was continued until 2 months after the maintenance dose of 6.6 g (= 200 mL) of cow’s milk protein was achieved [18]. Although all children in this setting reached the maintenance dose, 3 (60%) experienced renewed anaphylactic reactions 2.5 – 3.5 months after omalizumab discontinuation, with a decrease in the amount of still tolerated cow’s milk to 50 – 100 mL, requiring the continuation of AIT-accompanying omalizumab therapy. In another open-label study, 11 cow’s milk-allergic patients aged 7 – 17 years received 9 weeks of preceding treatment with omalizumab followed by omalizumab-accompanied oral AIT up to a daily dose of 1,000 mg of milk powder [19]. Nine (81.8%) patients achieved the target maintenance dose of 2,000 mg and also tolerated oral challenge with a maximum of 3,000 mg (cumulative 7,250 mg) of cow’s milk powder. In a DBPC study, 57 patients (7 – 32 years old) were treated with either omalizumab or placebo for 18 weeks and then received unblinded AIT with cow’s milk under this medication [20]. After 16 months, unblinding was followed by continuation of omalizumab administration in the actively treated group for 12 additional months. In the subsequent oral provocation test, more omalizumab-treated subjects (88%) than placebo-treated subjects (74%) subjects tolerated a cumulative dose of 10 g of cow’s milk powder, but this difference was not significant. However, the omalizumab patients required significantly fewer cow’s milk administrations for this purpose (198 vs. 225) and a shorter initiation period (25.9 vs. 30 weeks). Nevertheless, there was no significant difference between the two groups in the number of patients who lost their tolerance in the subsequent 4-week follow-up phase, or in the lower amount of cow’s milk still tolerated. 

Only case series are found on the effect of omalizumab administration in oral hen’s egg AIT. In one series, a new attempt of tolerance induction with chicken egg was performed in 9 children who previously had received an unsuccessful egg-AIT, after 9 weeks of pre-treatment with omalizumab and under its continued application until 2 months after a maintenance dose of 1.8 g hen’s egg protein (= about half an egg) was achieved [18]. Although all of them tolerated an oral provocation with 3.6 g (= 1 egg), 3 patients (33%) experienced a renewed loss of tolerance 2.5 – 4 months after discontinuation of omalizumab with a significant decrease in the amount of egg still tolerated, in some cases by more than 50%. Another study of 3 children with egg allergy showed a successful oral AIT after pre-administration of omalizumab for 2 – 3 months, but also in this case renewed allergic reactions to chicken egg after discontinuation of omalizumab were seen [21]. It remains unclear what influence the (possibly too short) duration of omalizumab treatment or insufficient maintenance therapy (at least half or one egg 3 × per week) had on this; moreover, the trial encompassed a very small number of patients, all of whom had already unsuccessfully discontinued previous AIT with hen’s egg, without otherwise clearly defined inclusion criteria. 

Some studies investigated the effect of omalizumab on AITs with multiple foods [22, 23, 24]. Since, on the one hand, about one third of all patients with food hypersensitivity are allergic to more than one food and sequential administration of AITs would impose a large time burden on them, and, on the other hand, simultaneous administration of multiple food allergens may increase the risk of anaphylactic reactions, such studies are of particular significance. In a phase I trial, 25 children and adolescents received oral AIT with up to 5 allergens (cow’s milk, egg, peanut, tree nuts, soy, wheat, and/or sesame) simultaneously after pretreatment with omalizumab for 8 weeks [22]. During the 10-week AIT, omalizumab was continued for 8 weeks. Here, 76% were brought to the target amount of 1,250 mg total food protein in the rush initiation phase; even with a slower initial dose increase, and all participants ultimately reached the maintenance dose of 4 g protein per allergen (i.e., up to a cumulative 20 g protein for 5 allergens). A DBPC phase II trial then evaluated 48 participants aged 4 – 15 years who received oral AIT with 2-5 food allergens [23]. Omalizumab (or placebo) was again applied initially for 8 weeks, then for an additional 8 weeks during the initiation of AIT, which was administered for a total of 20 weeks. At the end of the study, significantly more of the patients initiated on omalizumab tolerated 2 g protein of at least 2 food allergens (83% vs. 33%) or even at least 4 allergens (77% vs. 0%). Another open-label phase II trial, also with 8-week omalizumab pre-treatment followed by 8-week continuation under AIT, with up to 5 allergens applied simultaneously, which included also adults (age 5 – 22 years), showed similar results [24]. 

The majority of the studies presented thus demonstrate, in part impressively, the potential of omalizumab to achieve tolerance to food allergens in the context of oral AIT, also with regard to the single and cumulative or combined allergen doses achieved. The supportive effect of omalizumab here primarily refers to the period of its administration during allergen up-dosing, but not to the persistence of tolerance achieved after its discontinuation. Considering the influence of the anti-IgE antibody on the development of AIT-dependent side effects, the picture is also predominantly positive. For example, compared with placebo, the application of omalizumab resulted in a significant reduction in allergic reactions to 8.5% vs. 26.1% in the up-dosing phase and 0.7% vs. 14.4% in the maintenance phase of AIT with cow’s milk, which included both the lower and higher grades of severity [20]. In a DBPC study of peanut-allergic patients, there was a substantial decrease of moderate hypersensitivity reactions to 14.2% in the omalizumab cohort compared to 75% in the placebo group, while the differences were not as marked for mild (57.1 vs. 62.5%) or severe reactions (10.7 vs. 12.5%) [17]. Overall, application of omalizumab thus allows to significantly reduce the risk of allergic AIT reactions, but severe anaphylaxis requiring epinephrine treatment must still be expected [17, 19, 20, 22]. 

## Omalizumab in AIT with insect venoms

Insect venom allergies relevant in Germany involve allergic reactions to the venom of Hymenoptera, which include bees and wasps. Affected patients can suffer severe anaphylaxis, and successful tolerance induction to the eliciting insect venom by AIT is therefore of significant relevance [25]. However, hypersensitivity reactions may occur, especially during induction, which may also result in premature discontinuation of AIT. Promoting risk factors are, for example, co-morbidities such as bronchial asthma or mastocytosis as well as the venom used, since bee venom is often not so well tolerated [25]. Therefore, administration of omalizumab (temporarily) accompanying AIT with hymenoptera venom represents a promising therapeutic option, as demonstrated by exemplary case reports [26, 27, 28, 29, 30] and a retrospective case analysis [31]. 

The latter includes 10 patients with hymenoptera venom allergy severity grade II (1 patient) to III according to Ring and Messmer, of whom 5 had mastocytosis and 6 had elevated basal mast cell tryptase (> 11.4 µg/mL). Due to repeated systemic reactions (2 to more than 7 times) to the venom extract (8 × bee venom, 2 × wasp venom), which occurred for the first time after 1 – 90 (median 11) months of AIT and could not be influenced by the prophylactic administration of antihistamines or corticosteroids (2 patients), treatment with omalizumab was started. Under this, AIT was re-initiated until a maintenance dose was reached that was 100 µg above the previously not tolerated dose. After 8 – 31 (median 17) weeks, application of omalizumab was stopped, and a sting challenge was performed in 8 patients 6 – 39 months later under continued AIT, revealing that venom tolerance was achieved in all of the patients. One subject with mastocytosis and repeated later field stings experienced loss of tolerance with a systemic reaction severity grade I – II. 

Dose increases under AIT were well tolerated with concomitant omalizumab therapy, without systemic side effects, with all patients receiving additional administration of antihistamines (previously shown to be ineffective). Even after discontinuation of omalizumab, all but 1 patient, who after 2 years of complication-free AIT again showed systemic symptoms to the administration of bee venom (and also reacted to a field sting again, see above), continued to tolerate the venom injections, which were continued for a longer period of time in the majority of patients due to the individual risk profiles. In comparison, a control group of 5 patients with comparable side effects to AIT, also assessed retrospectively, all had to discontinue AIT without tolerance induction. In summary, application of omalizumab represents a promising option for insect venom-allergic patients with an increased risk profile and reduced tolerability of injected venom to achieve venom tolerance. Furthermore and in contrast to AIT with food, tolerance is long lasting even when omalizumab is only temporarily administered. Nevertheless, there are also patients who do not respond and still show hypersensitivity reactions during AIT; while the omalizumab dose in these cases was at maximum 300 µg/mL, this may not have been sufficiently high enough [30]. 

## Omalizumab in AIT: practical implementation 

There is no standard protocol of how omalizumab is administered to support successful AIT while minimizing complications. In principle, two different approaches are possible. First, the application can be performed according to the general recommendations for asthma treatment, i.e., adapted to total serum IgE levels and body weight [10, 29, 31]. The minimum effective dose is 150 µg/injection, which is why this can be chosen to start treatment [30, 32]. It corresponds to 100% of the dose applied in an asthma patient with serum IgE < 100 kU/L and body weight < 70 kg, or 50% of the dose treating chronic spontaneous urticaria. However, depending on the response, an increase to 300 µg/mL or even 450 µg/mL may be required to avoid AIT-dependent adverse reactions. Administration should start some time before (re)initiation of AIT, otherwise IgE reactivity may not yet have dropped sufficiently [30]. In the various studies and case reports, this period was chosen very variably and ranged from 1 to 16 weeks, with injections every 14 or 28 days (Table 1). While in asthma patients and food-allergic patients IgE and body weight-adapted doses and rather longer pre-treatment intervals were often selected, insect venom-allergic patients mostly received standardized doses of 150 or 300 µg/injection over an often shorter time window of 1 – 5 weeks. Subsequently, with the start of immunotherapy, omalizumab applications were continued for ~ 3 – 6 months in parallel with AIT at 4-week injection intervals. In individual cases, long-term continuation (over several years) may be required [29, 33]. Testing of successful tolerance induction is then performed several months after discontinuation of the anti-IgE antibody, for example, after 10 weeks for oral AIT [10] or 6 months for insect venom AIT [30, 34]. 

## Omalizumab in AIT: immunologic effects 

The main mechanism of action favoring the development of allergen tolerance in patients previously not tolerating AIT is the decreased IgE reactivity due to complexation of free allergen-specific IgE antibodies (Figure 1A), the associated reduction of receptor-bound IgE, and the subsequently decreased expression of FcɛRI on basophil granulocytes and mast cells as major effector cells of the allergic response [3, 4, 35]. Thus, administration of omalizumab prior to AIT initiation leads to a significant down-regulation of allergen-specific activation of basophils [36, 37]. 

Furthermore, omalizumab also leads to a decrease in the high affinity IgE receptor on dendritic cells and thus their ability of IgE-mediated allergen binding and successive activation of allergen-specific T cells [38]. It is therefore speculated that omalizumab is also therapeutically effective via T-cell effects. For example, asthma patients treated with the anti-IgE antibody were found to have a decrease in IL-13-secreting T helper cells [39], and in patients with chronic urticaria, the clinical response to omalizumab correlated with a decrease in IL-10-, IL-31-, and IFN-γ-secreting T lymphocytes [4]. In a study of peanut-allergic children, there was a significant decrease in allergen-specific T cells in the 12-week pre-treatment period with omalizumab prior to initiation of oral AIT [40]. This reduction involved both CD4+ helper T cells and Foxp3+ regulatory T cells. During AIT, their numbers returned to normal, but regulatory T cells lost their previously increased IL-4 expression. Although these data suggest a significant immunoregulatory T-cell-specific effect of omalizumab, these effects have not been demonstrated in other studies, as no significant changes in effector or regulatory T cells were seen in cow’s milk-allergic patients on AIT preceded by omalizumab administration or in another appropriately treated group of asthma patients [36, 37, 41]. Further studies utilizing for example sensitive functional T-cell assays and taking into account dose and duration of anti-IgE antibody therapy would be desirable. 

## IgG anti-allergen antibodies

Another interesting approach utilizing antibodies in AIT is the application of IgG anti-allergen antibodies. In this case, the target is not the IgE antibodies directed against an allergen, but the allergens themselves. The theoretical concept behind this approach is the AIT-mediated production of these antibodies in the course of successful tolerance induction. Here, IgG antibodies primarily of isotype IgG4 are able to bind the relevant allergen and thus prevent its IgE-mediated fixation on allergic effector cells such as mast cells, basophilic and eosinophilic granulocytes, and on antigen-presenting cells such as dendritic cells and B lymphocytes (Figure 1B). These immunoglobulins are therefore also referred to as allergen-blocking antibodies, which prevent both clinical reactions caused by the release of histamine, leukotrienes, and other mediators and the immunological pathophysiology of continued activation of allergen-specific T helper 2 cells, and their allergen blocking capacity is considered an important parameter of successful AIT [42, 43, 44]. 

That these IgG antibodies could also be directly used therapeutically was first demonstrated more than 40 years ago when purified immunoglobulins from beekeepers, who naturally develop high serum concentrations of bee venom-specific polyclonal IgG antibodies due to frequent sting events, mediated protection in bee venom-allergic patients to sting challenge-provoked anaphylaxis [45]. Recently, it was shown that two recombinantly produced IgG4 antibodies against different epitopes of the major cat allergen Fel d 1 provided protection against allergen-induced symptoms [46]. The two monoclonal antibodies exhibited a singular blocking capacity of ~ 50% in vitro and in a passive cutaneous anaphylactic mouse model, which increased to ~ 80% when co-administered. In a placebo-controlled phase 1b study of 73 cat-allergic patients, a single subcutaneous injection of 600 mg of a 1 : 1 mixture of these antibodies was administered, resulting in a significant reduction in allergic symptoms in nasal provocation test, that was still detectable even 85 days after their application (with an IgG half-life of ~ 21 days). 

Administration of epitope-specific IgG antibodies thus represents a promising approach, particularly for the treatment of seasonal respiratory allergies, since a single injection should be sufficient for effective protection during the pollen season. Allergic patients who primarily react to only 1 major allergen would be priority candidates, in order to avoid having to apply too many different antibodies, since more than 1 allergen epitope should be blocked for achieving an efficient therapeutic effect. So far, it is unclear whether repeated administration and thus persistent blockade of IgE-mediated allergen presentation to T cells could possibly also result in a long-term loss of allergy-specific T helper 2 activity, for example through T cell deletion or anergy, as is has been shown for AIT [42, 44]. In this case, this procedure could also substitute the longer-term effects of AIT. Further studies, focusing on immune mechanisms and the dynamics of different T-(helper and regulatory) cell activities as well as allergen-specific IgE concentrations would be of particular interest. 

## Conclusion

In summary, biologics in the form of monoclonal antibodies are therapeutically applicable at different levels of AIT. For example, anti-IgE antibodies may allow successful tolerance induction, particularly in patients at increased risk of systemic reactions to allergen administration in the setting of AIT. As the only approved antibody to date, omalizumab has shown in various studies that, based on the individual risk profile of those affected, higher allergen doses can be applied regularly, the rate of AIT-dependent adverse reactions can be minimized, and therapy compliance can be increased. However, increased local reactions are usually not prevented and AIT-related anaphylaxis cannot be completely ruled out by omalizumab treatment. In this context, the potential of newer developments and preparations such as the anti-IgE antibodies ligelizumab and quilizumab (which can deplete IgE-bearing plasmablasts and B cells), the DARPins (Designed Ankyrin Repeat Proteins) or the fusion proteins, which fuse the FcɛRI with the inhibitory FcγRIIb receptor, might be of particular interest (Figure 1C) [47, 48, 49]. Also, if appropriate, approaches at the T-cell level in the form of inhibition of T-helper 2 effects stimulating IgE synthesis, for example by administration of the IL-4/IL-13 receptor-blocking antibody dupilumab, could represent a therapeutic option. In addition, allergen-blocking IgG antibodies represent a potential alternative to AIT that can achieve effective symptom reduction, particularly in allergic patients with clinically relevant (mono)sensitization to a distinct major allergen, as demonstrated in a pilot study using Fel d 1-sensitized cat-allergic patients as an example [46]. In addition to more comprehensive clinical trials on these treatment modalities, more detailed elucidation of additional immunological effects, such as potential immunoregulatory impact on T lymphocytes, is an as yet insufficiently analyzed area of research, the investigation of which may open up further insights for the therapeutic use of biologics in AIT. 

## Funding

None. 

## Conflict of interest

Wolfgang Pfützner served on Advisory Boards of ALK-Abelló, received contributions on lectures for ALK-Abelló, Thermo Fisher, Cilag-Jansen, Novartis, and Lilly and funding for research projects from Thermo Fisher and Biomay. 

**Table 1.** Administration of omalizumab in allergen immunotherapy: practical implementation.

AIT	Dose (µg/injection)	Start before AIT (weeks)	Injection interval (weeks)	Continuation during AIT (weeks)	Injection interval (weeks)	Tolerance testing (weeks)
**Overview of all studies**	150 – 300 (450)*	1 – 16	2 or 4	12 – 24	4	8 – 24
**Examples**						
Aeroallergens [10]	150*	13	2 or 4	3	2	–
Food [16]	150*	12	2 or 4	8	2 or 4	10
Insect venom [31]	150*	5	2	16 (8 – 31)	4	24 (24 – 156)

*Starting dose usually according to recommendations for administration in bronchial asthma (150 µg/injection for body weight < 70 kg, total IgE < 100 kU/l), increasing if necessary.
